# Aneurysm of the Aorta

**Published:** 1901-11-09

**Authors:** 


### ANEURYSM OF THE AORTA.

Dr. Whipham,[1] medical registrar St. George's Hospital, records a remarkable case of aneurysm of 16 years' duration, which ended by rupture externally. In 1884 the patient began to suffer from incessant cough and thoracic pains, and in 1886 a swelling was noticed on the right side of the sternum. He was then admitted to St. George's, when there was a rounded, regular, smooth tumour, 3½ inches in diameter, occupying the first and second right interspaces and the adjacent edge of the sternum. It was expansile, but no bruit was heard over it, though one was present in the right subclavian and carotid arteries. The voice was hoarse, the cough was suggestive of pressure, and there was inspiratory stridor. During his first three weeks in hospital the aneurysm increased rapidly in size till it eroded the whole of the upper part of the sternum, measuring 6¼ by 4½ inches. Two eminences appeared on the surface and the skin over the tumour was tense, shiny, and discoloured. The patient was described at this time as being "weak, ill, and hopeless," and it is an interesting thing to note that, notwithstanding his apparently desperate condition, he began gradually to mend, the tumour steadily decreased in size and seemed to consolidate, while the prominences disappeared. After lying in bed for eight months the patient got up again, the pulsating area then measuring only 1½ by 1¼ inch, and he was discharged in January, 1887. The treatment adopted had consisted of scruple to ½-drachm doses of potassium iodide, with morphine for the relief of pain, while the fluid part of his diet was restricted to a pint in the 24 hours. A year after his discharge he returned to work, which, however, did not involve manual labour. In 1892, after a violent cold with cough, he had violent shooting pains in the neck and left arm, and a rapid bulging of the aneurysm at the episternal notch. He was again admitted suffering from such dyspnœa and dysphagia that his condition was regarded as very serious. Again the iodide treatment was resumed, with the result that he much improved, and was discharged. In 1895 he was admitted with pleurisy and pneumonia. From this time the aneurysm became markedly worse, but he continued to work on and off until the beginning of 1899. There was still no bruit over the aneurysm, and no pressure symptoms beyond slight stridor and a brassy cough. He was admitted again, and for the last time in February, 1901, the aneurysm then having increased to the size of the closed fist, and presenting three lateral projections. From a small ulcerated surface, where pulsation was marked, there oozed a small quantity of blood. This superficial ulceration gradually extended. On April 9th the patient had a rigor, and sixty hours later a large conical mass of laminated fibrin, the whole of the superficial part of the aneurysm, came away leaving a considerable cavity in the chest in which no definite pulsation could be seen. For nine days the patient continued fairly comfortable when the aneurysm suddenly burst externally, and he died in about a quarter of an hour. The length of time which this aneurysm lasted, even after it had become well defined, was remarkable. The case well illustrates the very much better chance a man has when such an aneurysm extends forwards than when it takes an opposite direction, when of course pressure effects are inevitable ; it also shows how gradually the end may come when rupture takes place externally, compared with the sudden death which internal rupture causes.

[1] Brit. Med. Jour., Nov. 2.

